# Development of finely tuned liposome nanoplatform for macrophage depletion

**DOI:** 10.1186/s12951-024-02325-7

**Published:** 2024-02-29

**Authors:** Tae Hyeon Choi, Ran Ji Yoo, Ji Yong Park, Ji Yoon Kim, Young Chan Ann, Jeongbin Park, Jin Sil Kim, Kyuwan Kim, Yu Jin Shin, Yong Jin Lee, Kyo Chul Lee, Jisu Park, Hyewon Chung, Seung Hyeok Seok, Hyung-Jun Im, Yun-Sang Lee

**Affiliations:** 1 Department of Nuclear Medicine, Seoul National University College of Medicine, Seoul, South Korea; 2 Department of Molecular Medicine and Biopharmaceutical Sciences, Graduate School of Convergence Science and Technology, Seoul National University, Seoul, 08826 South Korea; 3 Department of Nuclear Medicine, Seoul National University Hospital, 101 Daehak-Ro, Jongno-Gu, Seoul, South Korea; 4 Biomedical Research Institute, Seoul National University Hospital, Seoul, South Korea; 5 Institute of Radiation Medicine, Medical Research Center, Seoul National University College of Medicine, Seoul, South Korea; 6 School of Dentistry, Seoul National University, Seoul, South Korea; 7 Cancer Research Institute, Seoul National University College of Medicine, Seoul, South Korea; 8 Department of Biomedical Sciences, Seoul National University College of Medicine, Seoul, South Korea; 9 Division of Applied RI, Korea Institute of Radiological and Medical Sciences (KIRAMS), Seoul, South Korea; 10 Department of Microbiology and Immunology, and Institute of Endemic Disease, Seoul National University College of Medicine, Seoul, South Korea

**Keywords:** Liposome, Macrophage, Clodronate, Molecular imaging, Click chemistry

## Abstract

**Background:**

Immunotherapy with clodronate-encapsulated liposomes, which induce macrophage depletion, has been studied extensively. However, previously reported liposomal formulation-based drugs (Clodrosome^®^ and m-Clodrosome^®^) are limited by their inconsistent size and therapeutic efficacy. Thus, we aimed to achieve consistent therapeutic effects by effectively depleting macrophages with uniform-sized liposomes.

**Results:**

We developed four types of click chemistry-based liposome nanoplatforms that were uniformly sized and encapsulated with clodronate, for effective macrophage depletion, followed by conjugation with Man-N_3_ and radiolabeling. Functionalization with Man-N_3_ improves the specific targeting of M2 macrophages, and radioisotope labeling enables in vivo imaging of the liposome nanoplatforms. The functionalized liposome nanoplatforms are stable under physiological conditions. The difference in the biodistribution of the four liposome nanoplatforms in vivo were recorded using positron emission tomography imaging. Among the four platforms, the clodronate-encapsulated mannosylated liposome effectively depleted M2 macrophages in the normal liver and tumor microenvironment ex vivo compared to that by Clodrosome^®^ and m-Clodrosome^®^.

**Conclusion:**

The newly-developed liposome nanoplatform, with finely tuned size control, high in vivo stability, and excellent ex vivo M2 macrophage targeting and depletion effects, is a promising macrophage-depleting agent.

**Graphical Abstract:**

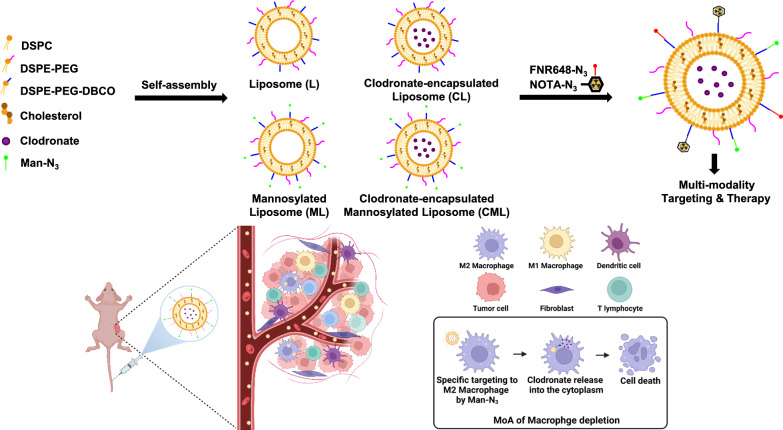

**Supplementary Information:**

The online version contains supplementary material available at 10.1186/s12951-024-02325-7.

## Introduction

Inflammation is the response of the immune system to external pathogens, irradiation, and toxic compounds. These defense mechanisms activate and promote the accumulation of immune cells [1–3]. Among the immune cells, macrophages play a significant role in the inflammatory responses. Macrophages are myeloid immune cells that orchestrate various aspects of immunity. Macrophages are present in various tissues of the body, and they can polarize into other subtypes, such as M1 and M2 macrophages, depending on their environment. The representative roles of M1 and M2 macrophages include pro- and anti-inflammatory responses, respectively. Macrophage dysfunction (such as in the case of tumor-associated macrophages (TAMs)) contributes to inflammation progression and tumorigenesis [3–6]. Therefore, research on macrophage depletion is aimed at inflammatory disease treatment and immune checkpoint therapy [7–9]. Typically, anti-PD-1 or PD-L1 immune checkpoint blockade is known to induce M1 macrophage polarization, which has anti-tumor effects [10]. Mannose ligands are extensively used to selectively target the mannose receptors on the surface of macrophages, for enhancing the effectiveness of macrophage depletion [11–13].

Clodronate is a first-generation bisphosphonate and an active pharmaceutical ingredient (API) used in the clinical treatment of inflammatory diseases such as rheumatoid arthritis and osteoarthritis [14, 15]. It is encapsulated in liposomes and used to deplete macrophages [15–17]. Following the phagocytosis of clodronate-encapsulating liposomes by macrophages, the phospholipid bilayer is disrupted by lysosomal phospholipase. Subsequently, the clodronate is released intracellularly, causing cell death via irreversible functional damage and apoptosis [17–19].

Various nanostructure-based pharmaceutical formulations, such as liposomes [20, 21], albumins [22], antibodies [23], exosomes [24], and iron oxide nanoparticles [25], have been studied extensively. Among them, liposomes are versatile nanocarriers that have the advantage of being biocompatible, non-toxic, non-immunogenic, and biodegradable. The amphiphilic phospholipid bilayer structure of liposomes is similar to that of mammalian cell membranes; therefore, liposomes can be effectively used for cellular uptake, owing to the cell-to-liposome interactions. The ability of liposomes to encapsulate hydrophilic (polar) and hydrophobic (nonpolar) APIs can reduce their toxic effects and improve their circulation half-life by preventing drug degradation [26–29]. Therefore, liposomes are used as drug delivery system (DDS) nanoplatforms to effectively deliver clodronate.

Commercially available liposomal formulation-based drugs for macrophage depletion include Clodrosome^®^ and m-Clodrosome^®^ (Encapsula Nano Sciences, TN, USA). They are administered through various routes in studies on inflammatory diseases [30–33]. However, the hydrodynamic diameter of Clodrosome^®^ and m-Clodrosome^®^ is relatively non-uniform at 512.5 ± 390.7 nm and 904.4 ± 216.5 nm, respectively. The particle size of liposomes influences hepatic uptake, pharmacokinetics, biodistribution, tissue diffusion, kidney excretion, and blood circulation time when injected into the body, so the therapeutic effect may not be consistent [27, 28]. Therefore, controlling and validating parameters such as mean diameter and polydispersity index (PDI) are crucial for the clinical application of liposomal formulation-based drugs [34].

Among the TAMs in the tumor microenvironment (TME), M2 macrophages promote tumor growth [35]. Therefore, we developed a macrophage-depleting agent that can control TME by specifically targeting M2 macrophages, which are TAMs, and effectively reducing their numbers.

The goal of this study was to demonstrate that size-consistent liposomal nanoplatforms effectively deplete macrophages, laying the foundation for future immune checkpoint therapy. In this study, we developed a click chemistry-based liposome nanoplatform that was uniformly sized and suitable for the encapsulation of clodronate for effective macrophage depletion. Click chemistry can be used as a surface modification tool, because liposomes maintain their intrinsic properties during functionalization via site-specific conjugation. We used a strain-promoted alkyne-azide cycloaddition (SPAAC) reaction, which is not only copper-free and has a fast reaction rate, but also compatible and bio-orthogonal in vivo [36–40]. To further explore the efficacy of the liposomes, we synthesized four different types of liposomes and confirmed their superiority through histological and efficacy evaluations. We believe that our liposome nanoplatform is superior in its macrophage depletion effect compared to commercialized Clodrosome^®^ and m-Clodrosome^®^.

## Results and discussions

### Liposome nanoplatform characterization

**Fig. 1:**
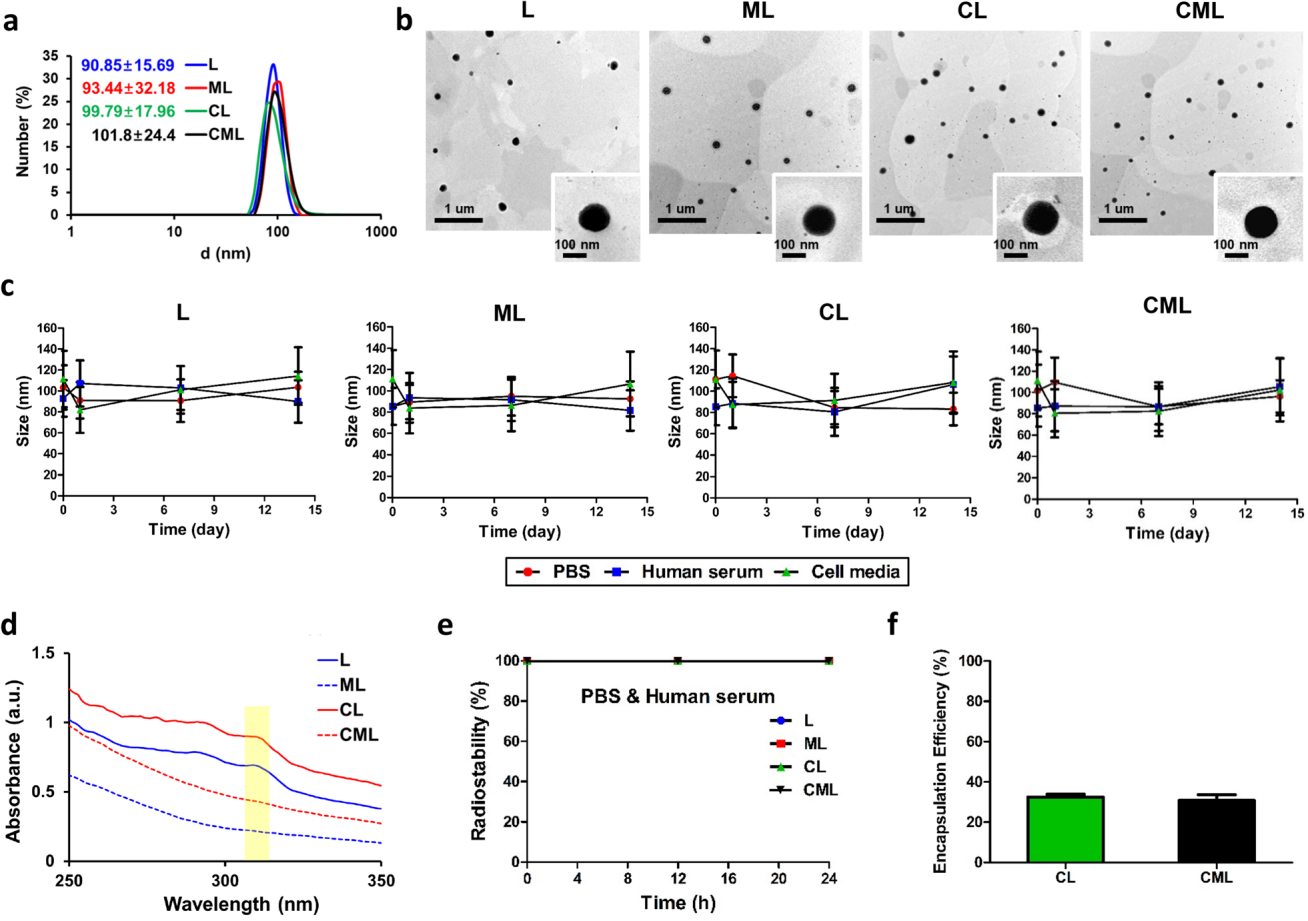
Characterization of the liposome nanoplatform for macrophage depletion **a** Hydrodynamic diameter of liposomes. All data were averaged from five measurements using the DLS system. **b** TEM images of liposome nanoplatform with low and high magnifications (down). **c** Stability test of liposome nanoplatform in physiological solutions (PBS, human serum, and cell media) for 14 days. **d** UV spectrum of peak change according to binding of Man-N_3_ to liposomes **e** Radiostability test in PBS and human serum at varying time points (0, 12 and 24 h) following radiolabeling through click chemistry. (f) Clodronate encapsulation efficiency of CL and CML (n = 3, mean ± SD). *P < 0.05, **P < 0.01

Doxil^®^, the first FDA-approved liposomal drug, and all other FDA-approved and currently investigated liposomal drugs are designed at 100 nm diameters [28, 41]. This is because most therapeutic liposomes are designed to avoid the mononuclear phagocytic system (MPS) uptake and to increase blood circulation time [27]. In addition, nanoparticles larger than 100 nm cannot pass through hepatocytes because the hepatic fenestration of the endothelium is approximately 100 nm [42, 43] Therefore, we synthesized a 100 nm liposome nanoplatform. The hydrodynamic diameters of the liposomes (liposome [L], mannosylated liposome [ML], clodronate-encapsulated liposome [CL], and clodronate-encapsulated mannosylated liposome [CML]) were 90.85 ± 15.69, 93.44 ± 32.18, 99.79 ± 17.96, and 101.8 ± 24.4 nm, respectively (Fig. 1a). All liposomes had a PDI of approximately 0.2, suggesting that they were a homogenous population [34]. The zeta potential tended to be slightly higher for Man-N_3_ binding via click chemistry; however, the results were within the margins of error. On the contrary, Clodrosome^®^ and m-Clodrosome^®^ had a large standard deviation in size, and their PDI was 0.543 and 0.461, respectively. This suggests that the populations were relatively heterogeneous (Table 1). These tendencies were also confirmed by the formation of a single peak for all liposomes, whereas multiple peaks were formed in the NTA analysis for Clodrosome^®^ and m-Clodrosome^®^ (Additional file 1: Fig. S2). The liposome nanoplatform had a spherical shape and uniform size distribution, as observed using transmission electron microscopy (TEM) (Fig. 1b). Liposome stability was assessed in various physiological solutions (phosphate buffered saline [PBS], human serum, and cell media) to determine the feasibility of in vivo utilization. The hydrodynamic diameters of the liposomes were maintained within the 20% error margin for 14 days (Fig. 1c). In addition, they showed no visible aggregates or precipitates for 14 days (Additional file 1: Fig. S2). The UV–visible spectrum shows peak intensities at specific wavelengths for DBCO (peak intensity at 309 nm, yellow square box) (Fig. 1d). Therefore, dibenzocyclooctyne (DBCO), a click chemistry derivative, adheres to the liposome surface. The radiochemical stabilities were over 95% for up to 24 h in PBS and human serum, indicating that in vivo utilization of the liposome nanoplatform would be effective (Fig. 1e). The clodronate encapsulation efficiency did not differ between CL and CML (Fig. 1f). In further experiments, the clodronate encapsulation efficiency did not significantly decrease, even 30 days after synthesis of the liposomes (CL and CML), indicating that clodronate was released from the liposomes in a sustained manner (Additional file 1: Fig. S3).

**Table 1:** Size and zeta potential of samples

Sample	Size (nm)	Poly dispersity index (PDI)	Zeta potential (mV)
L	90.85 ± 15.69	0.114	− 23.6 ± 8.06
ML	93.44 ± 32.18	0.221	− 21 ± 7.43
CL	99.79 ± 17.96	0.196	− 21.3 ± 4.72
CML	101.8 ± 24.4	0.239	− 21 ± 5.04
Clodrosome	512.5 ± 390.7	0.543	− 5.86 ± 5.13
m-Clodrosome	904.4 ± 216.5	0.461	− 3.12 ± 3.86

### In vitro cell viability test of the liposome nanoplatform for macrophage depletion

**Fig. 2:**
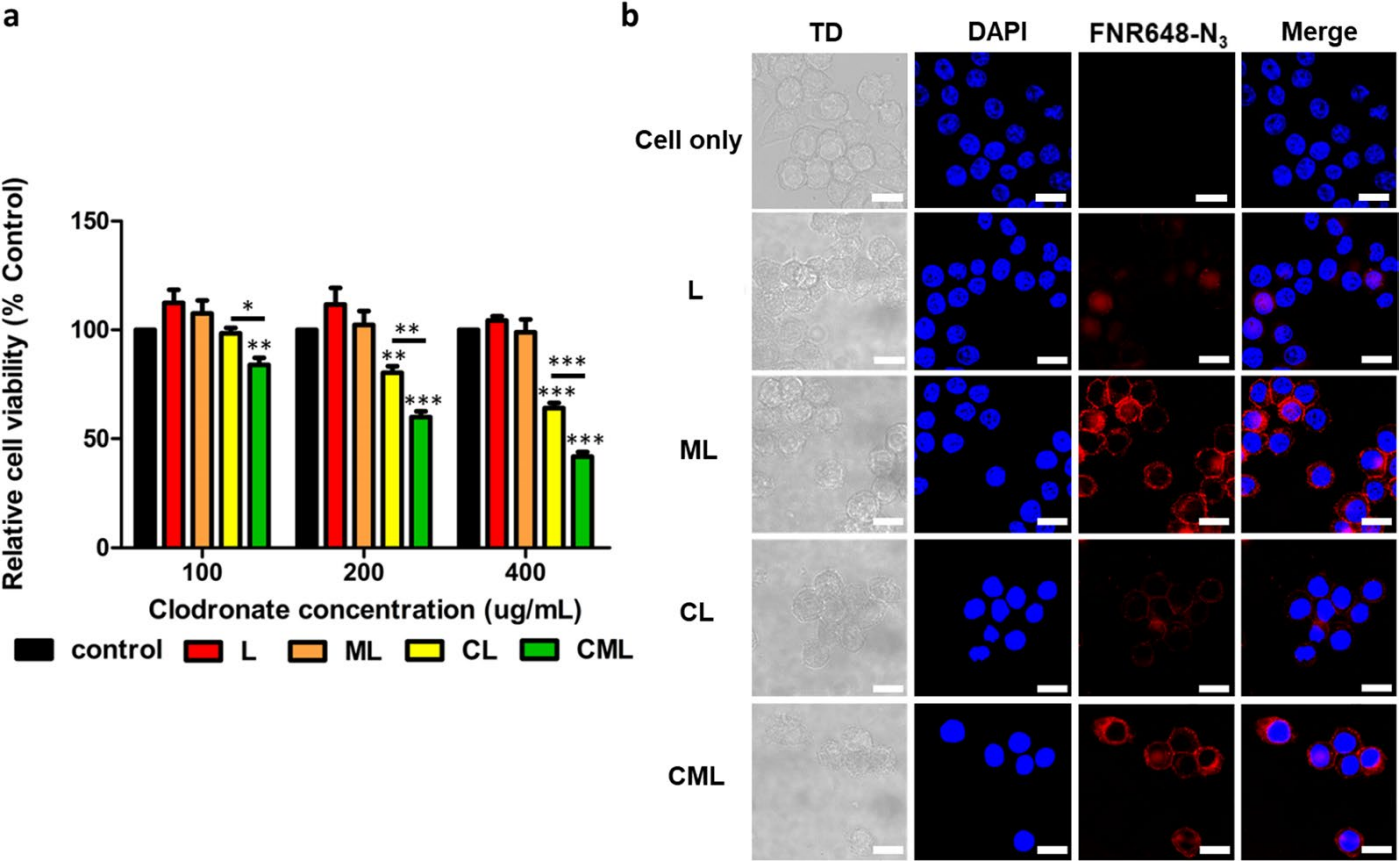
Cell viability and cellular uptake of liposome nanoplatform for macrophage depletion (**a**) Cell viability test was performed using a CCK assay with RAW264.7 cells at different concentrations of clodronate. (n = 3, mean ± SD). *P < 0.05, **P < 0.01, ***P < 0.001 (**b**) In confocal microscopy, mannosylated liposomes (ML and CML) were internalized in RAW264.7 cells, while L and CL showed minimal internalization. All scale bars in the images are 75 µm. TD: transmitted light channel, blue: nuclei (DAPI), red: fluorescence conjugated liposomes (FNR648-N_3_)

A cell viability test of the liposome nanoplatform was performed to determine toxicity at the cellular level (Fig. 2a). All experiments were performed using RAW264.7 cells. Clodronate-free liposomes (L and ML) did not alter cell viability compared to that in the control group. This indicates that the synthesized liposomes are reasonably biocompatible. In contrast, CL and CML decreased cell viability in a dose-dependent manner. This tendency was significantly higher in CML than that in CL at clodronate concentrations of 100, 200, and 400 µg/mL (P < 0.05, P < 0.01, and P < 0.001, respectively). The liposomes did not show significant differences in terms of cytotoxicity, when compared to Clodrosome^®^ and m-Clodrosome^®^ (Additional file 1: Fig. S4).

### In vitro cell uptake of liposome nanoplatform for macrophage depletion

The cellular uptake of the liposome nanoplatform was observed as red fluorescence images; these were used to assess the degree of specific binding to RAW264.7 cell (Fig. 2b). When we investigated the effects of liposomes on cellular uptake at several time points (0.5, 1, 2, 4, and 24 h), the most efficient cellular uptake was achieved at 4 h (Additional file 1: Fig. S5). The fluorescence signals of ML and CML were significantly higher than those of mannose-free liposomes (L and CL). This result implied that the MLs were specifically bound to the cells through the targeting ligand mannose. RAW264.7 cells did not exhibit autofluorescence in the FNR648-N_3_ wavelength band (Ex/Em: 648/663 nm), as observed through the absence of red fluorescence in cells not treated with liposomes.

### Pharmacokinetics and ex vivo biodistribution of the liposome nanoplatform

**Fig. 3:**
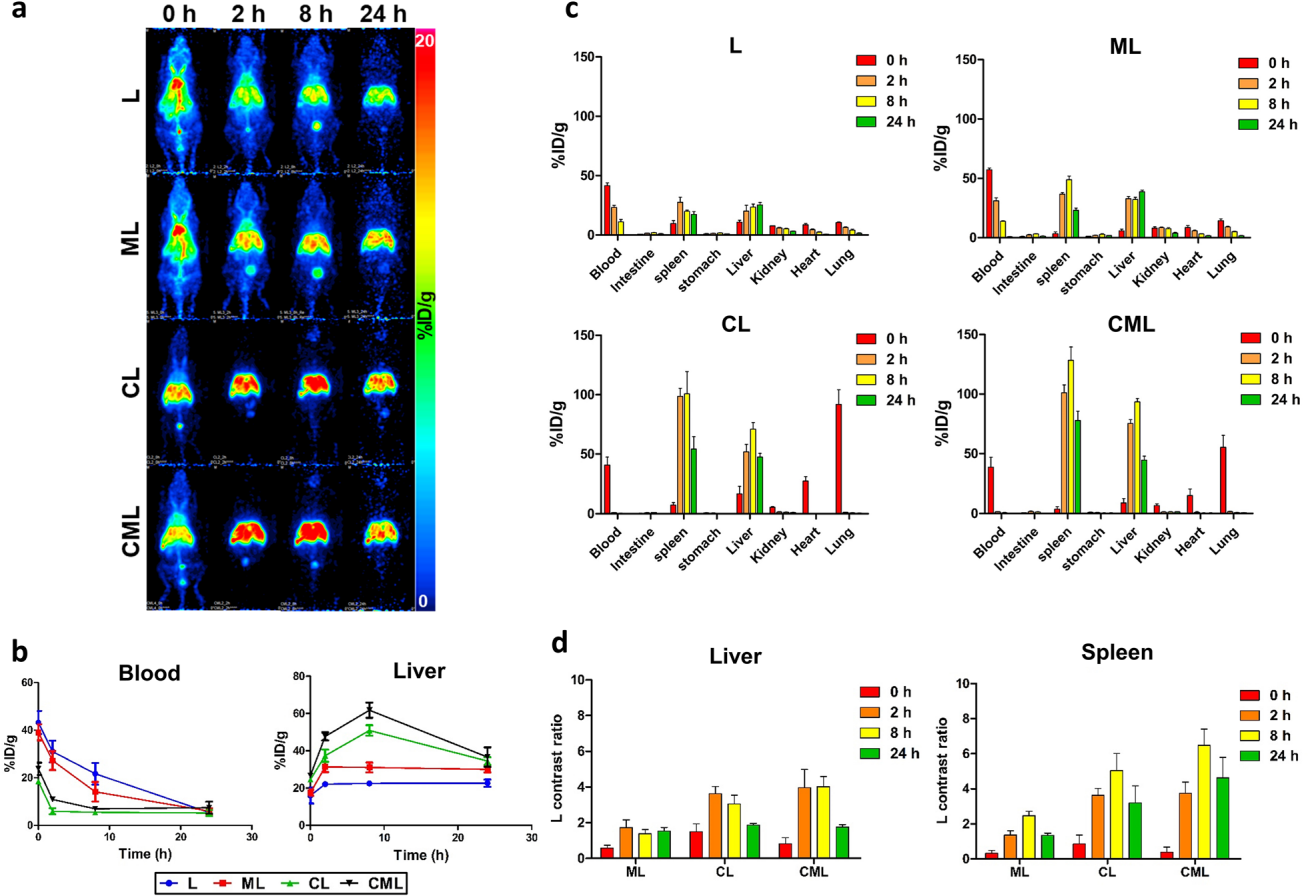
in vivo PET imaging and quantitative analysis of Liposome nanoplatform for Macrophage depletion (**a**) Representative PET images of normal mice (n = 3) at the different time points (0, 2, 8, and 24 h) after tail vein injection of ^64^Cu labeled liposomes (L, ML, CL, and CML). **b** Time activity curve of the blood pool and liver (n = 3, mean ± SD). **P < 0.01, ***P < 0.001 **c** Quantitative analysis of liposomes in various organs of normal mice, expressed as % ID/g (n = 4, mean ± SD). **d** Comparison of relative uptake of liposomes in the liver and spleen (n = 4, mean ± SD)

We performed biodistribution studies of the liposome nanoplatforms using positron emission tomography (PET) imaging to determine changes in the distribution over time. The biodistribution of the liposomes substantially differed following clodronate treatment (Fig. 3a). CL and CML had a shorter blood circulation time than L and ML at all time points; however, their liver uptake increased. In addition, ML and CML showed higher and longer liver uptake at all time points than L and CL. All experimental groups had a weak signal in the gallbladder, indicating that they are internalized by Kupffer cells [44]. Kupffer cells are liver-resident macrophages that play an important role in innate immune responses; they are located in the lumen of the liver sinusoid. The Kupffer cells constitute the MPS; therefore, they participate in liver metabolic functions by efficiently phagocytizing liposomes that enter sinusoidal blood [45–47]. In addition, nanoparticles smaller than 100 nm can pass through hepatocytes because of the endothelium size of hepatic fenestrations [42, 43]. Therefore, the liposomes are internalized into Kupffer cells rather than hepatocytes. These biodistribution tendencies appeared in the time-activity curve, quantitatively analyzed based on the PET images (Fig. 3b and Table 2). At 0 h and 8 h in the blood pool, L was the highest at 43.23 ± 4.84% ID/g and 21.67 ± 4.57% ID/*g*, respectively, which was two to four times higher than the values for CL and CML at the same time points. CML showed the highest liver uptake among the liposomes. It was 61.78 ± 4.06% ID/g at 8 h, which was 2.7, 2.0, and 1.2 times higher than that for L, ML, and CL at the same time points, respectively. The blood half-lives of L (174.2 min), ML (83.3 min), CL (4.7 min), and CML (5.5 min) were calculated using uptake of the blood pool using nuclear medicine imaging (Table 2). These results will serve as the basis for the functional evaluation of immunosuppressive drugs, through changes in the number of Kupffer cells, which are macrophages in the liver. The biodistribution of the liposome nanoplatform was confirmed using an ex vivo biodistribution analysis. CL and CML accumulated in the liver and spleen. They exhibited similar increases in splenic uptake over time, with the highest increase observed at 8 h. This tendency was also observed for liver uptake (Fig. 3c). This could be because the liver and spleen are sensitive to blood vascularization and contain various types of tissue-resident macrophages [41, 48]. In particular, CL and CML are irreversibly sequestered by MPS due to the clodronate API; therefore, they seem to be highly accumulated in MPS (the liver and spleen) [49]. The tendency of the liver and spleen to take up more CL and CML remained constant, when comparing the % ID/g of L with the rest of the experimental groups (Fig. 3d). Therefore, we aimed to prove the effectiveness of the liposome nanoplatform in macrophage depletion in vivo.

**Table 2:** Organ uptake of liposomes quantified using PET imaging

		Blood pool (% ID/g)		Liver (% ID/g)	
		Average	SD	Average	SD
L	0 h	43.23	4.84	15.97	4.24
	2 h	31.01	4.57	22.09	1.55
	8 h	21.67	4.57	22.50	1.27
	24 h	5.37	0.97	22.61	1.96
ML	0 h	39.10	3.37	17.52	1.59
	2 h	27.21	3.97	31.38	2.75
	8 h	14.08	4.12	31.04	2.64
	24 h	5.96	1.26	30.02	1.64
CL	0 h	18.45	0.72	24.58	1.38
	2 h	5.88	1.27	37.35	3.28
	8 h	5.46	1.00	50.90	2.80
	24 h	5.18	1.26	34.50	2.10
CML	0 h	23.75	2.62	26.72	1.36
	2 h	10.89	0.95	47.73	2.17
	8 h	6.93	0.53	61.78	4.06
	24 h	7.43	2.50	36.63	5.19

(n = 3 for each group)

### Evaluation of the immunological function of the liposome nanoplatform for macrophage depletion in liver tissue ex vivo

**Fig. 4:**
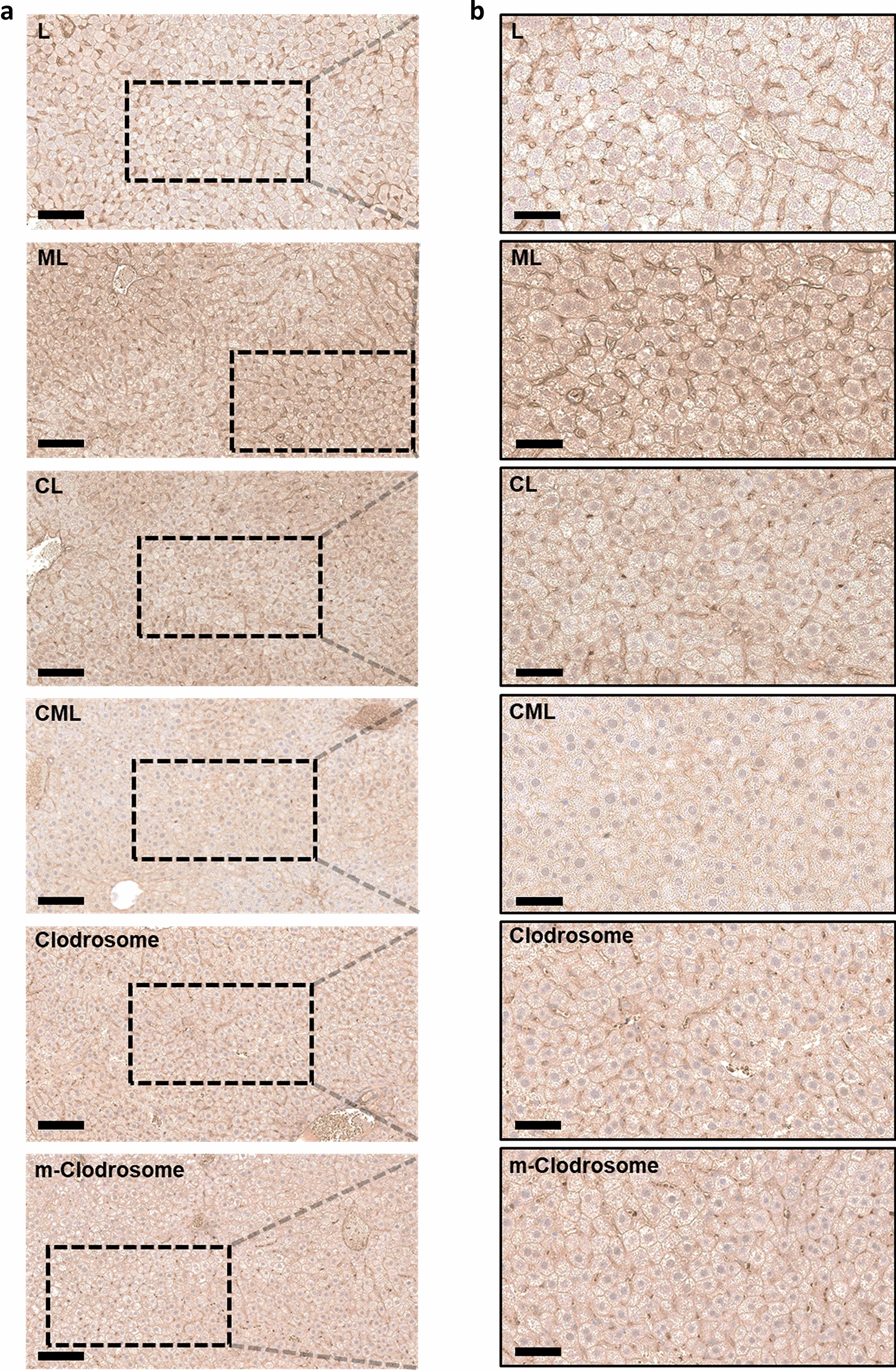
M2 macrophage populations in liver tissues imaged using anti-CD206 antibody (**a**) Immunohistochemistry stained images of liver tissues from normal mice injected with liposome nanoplatform. Scale bar = 100 μm. **b** Higher magnification images of the black-outlined area. Scale bar = 50 μm

To determine whether in vivo-injected liposomes effectively depleted macrophages in liver tissues compared to those in positive controls (Clodrosome^®^ and m-Clodrosome^®^), we dissected liver tissues and performed histological evaluations. Green fluorescence images from confocal microscopy were used to compare macrophage depletion (Additional file 1: Fig. S6). The green fluorescence signal of CML was the lowest among all the experimental groups, including the positive controls, indicating that it caused the highest depletion of macrophages in the liver tissue. Immunohistochemistry was performed using an anti-CD206 antibody as a M2 macrophage marker for histological analysis [10]. The expression level of the M2 macrophage surface marker CD206 was the lowest in the CML group among all experimental groups (Fig. 4). This can be attributed to two factors. First, mannose receptor expression increases in M2 macrophages [45]. The mannose receptor contains a carbohydrate recognition domain 4 (CRD_4_), and mannosylated nanoparticles can be specifically internalized by M2 macrophages because of the high-affinity binding between CRD_4_ and mannosylated nanoparticles [13]. Therefore, as the number of mannose receptors increases, more mannosylated nanoparticles can be internalized by M2 macrophages via increased CRD_4_. Second, the CML was uniform at 100 nm, but the positive controls were more than 500 nm in particle size and nonuniform. Kupffer cells phagocytose nanoparticles mainly via clathrin-mediated endocytosis when internalized via mannose receptors. Clathrin-mediated endocytosis internalizes particles in the size range of approximately 100 − 350 nm. In addition, Kupffer cells phagocytize nanoparticles through macropinocytosis, which internalizes particles in the range of 0.5 − 5 µm; however, macropinocytosis is rare [45, 50]. In other words, CML is efficiently internalized by the Kupffer cells via clathrin-mediated endocytosis, unlike the positive controls. Therefore, the histological results could be explained by the efficient internalization of CML, which has a constant size of 100 nm, through clathrin-mediated endocytosis into M2 macrophages, through the overexpressed mannose receptors on the surface. This induces clodronate-mediated apoptosis that results in M2 macrophage depletion. Hematoxylin and eosin (H&E) staining was performed to confirm that liposomes caused histological damage to the liver (Additional file 1: Fig. S7). There was no significant difference in the shapes of the nucleus and cytoplasm of hepatocytes in any of the experimental groups compared to that in the control group. Therefore, the liposomes are not hepatotoxic.

### Efficacy evaluation of liposome nanoplatform for macrophage depletion in TME ex vivo

**Fig. 5:**
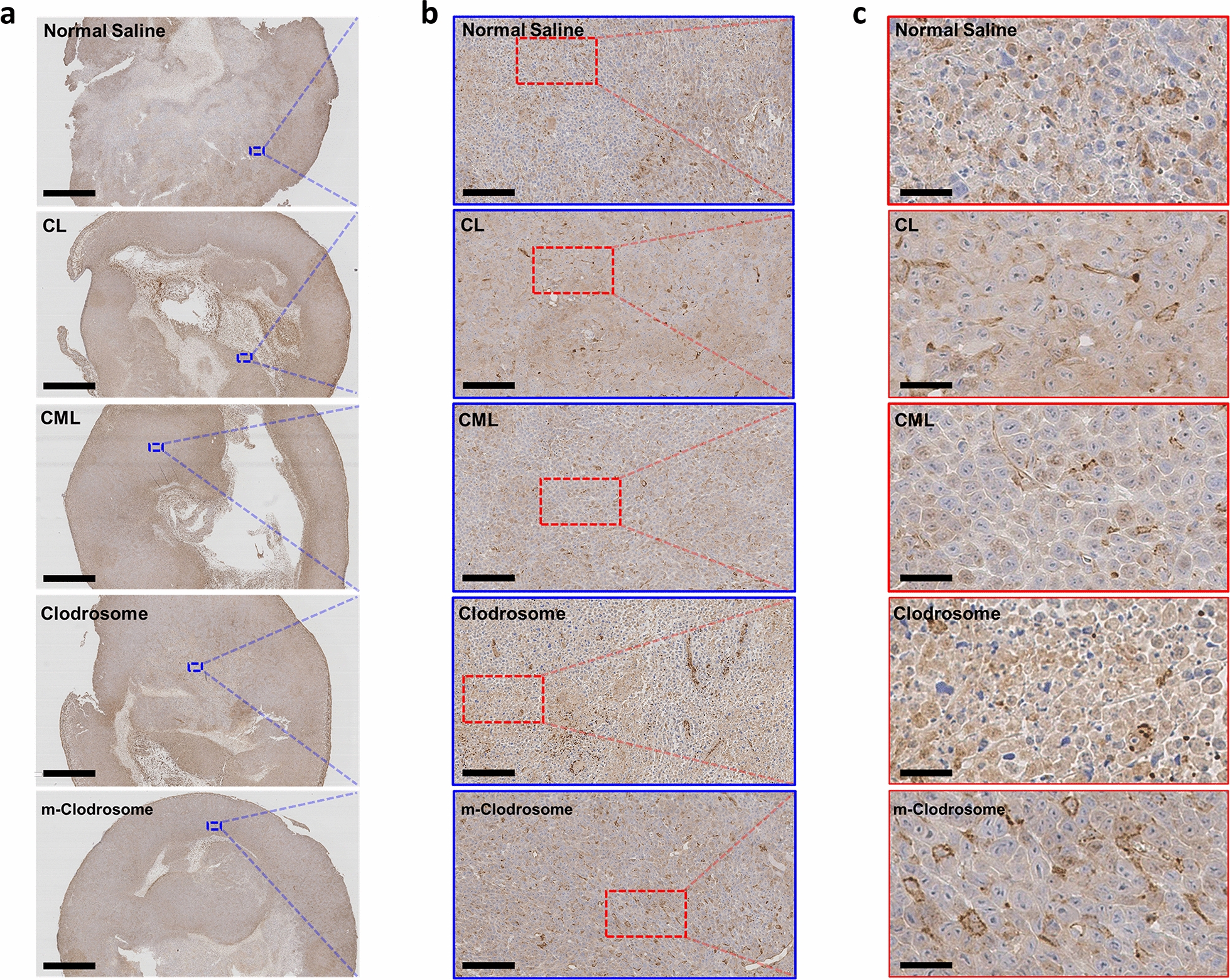
M2 macrophage populations in TME imaged using anti-CD206 antibody (**a**) Immunohistochemistry stained images of tumor tissues from 4T1-bearing mice injected with liposome nanoplatform. Scale bar = 2000 μm. **b** Higher magnification images of the blue-outlined area. Scale bar = 100 μm. **c** Higher magnification images of the red-outlined area. Scale bar = 25 μm

To confirm the efficacy of the liposome nanoplatform for macrophage depletion in TME compared to that in positive controls (Clodrosome^®^ and m-Clodrosome^®^), we used 4T1 breast cancer cells. M2 macrophage, a TAM among immune cells, plays an important role in tumor growth, metastasis, and angiogenesis in the TME of breast cancer [34]. We histologically evaluated the 4T1 tumor tissues using anti-CD206 antibody as an M2 macrophage marker. CML blocked tumor progression most efficiently, as indicated by the frequency of M2 macrophages (brown color) and the degree of hematoxylin staining of the nuclei (blue color) in the cancer cells (Fig. 5). Therefore, CML efficiently achieved specific targeting of M2 macrophages among the TAMs in the TME, and it caused macrophage depletion by inducing apoptosis with clodronate encapsulated in CML.

However, our liposome nanoplatform has some limitations; 100 nm may not be the optimal size for macrophage depletion because we evaluated the function of the liposome nanoplatform as a macrophage-depleting agent only for one size. Therefore, in future studies, the macrophage depletion effects of liposome nanoplatforms of various sizes should be evaluated.

## Conclusions

We developed a liposome nanoplatform for effective macrophage depletion. Our liposome nanoplatform exhibited (1) finely tuned size control, (2) high in vivo stability, and (3) excellent ex vivo M2 macrophage targeting and depletion effects. CML is superior to the already commercialized Clodrosome^®^ and m-Clodrosome^®^ for macrophage depletion.

Immune checkpoint inhibitors (ICIs) have been investigated over the last several years as tools for immune checkpoint therapy. However, ICIs show a low response rate of 15 − 30% in the case of solid tumors; therefore, they do not provide effective treatment to a large percentage of patients [51]. Combination therapy using ICIs and the liposome nanoplatform developed in this study, which can modulate the TME, could improve the therapeutic efficacy of ICIs, by effectively penetrating the TME and enhancing their interaction with TAMs. Therefore, this liposome nanoplatform could be a promising alternative for cancer immunotherapy.

## Methods and materials

### General

Distearoyl phosphatidylcholine (DSPC), cholesterol, 1,2-distearoyl-sn-glycero-3-phosphoethanolamine(methoxy(polyethylene glycol)-2000) (DSPE-PEG(2 k)), and 1,2-distearoyl-sn-glycero-3-phosphoethanolamine-N-[dibenzocyclooctyl(polyethylene glycol)-2000] (DSPE-PEG(2 k)-DBCO) were purchased from Avanti Polar Lipids Inc. (Alabama, USA). Disodium clodronate tetrahydrate was purchased from Tokyo Chemical Industry Co. Ltd. (Tokyo, Japan). Clodrosome^®^ and m-clodrosome^®^ were purchased from Encapsula Nano Sciences (Nashville, TN, USA). 2,2ʹ,2″-(2-(4-(3-(3-azidopropyl)thioureido)benzyl)-1,4,7-triazonane-1,4,7-triyl)triacetic acid (NOTA-N_3_) and azido-Flamma 648 (FNR648-N_3_) were purchased from FutureChem (Seoul, Korea). 1,1ʹ-Dioctadecyl-3,3,3ʹ,3ʹ-tetramethylindocarbocyanine perchlorate (DiI) was obtained from Invitrogen (Carlsbad, USA). 1-*O*-(2-(2-(2-azidoethoxy)ethoxy)ethoxy)-alpha-D-mannopyranoside (Man-N_3_) was purchased from Iris Biotech GmbH (Marktredwitz, Germany). Dulbecco modified eagle medium (DMEM), Roswell Park Memorial Institute 1640 (RPMI-1640), Fetal bovine serum (FBS), antimycotics/antibiotics (AA) were obtained from Gibco (Grand Island, NY). All other reagents and chemicals were purchased from Sigma-Aldrich (St. Louis, MO). A size-exclusion PD-10 column was purchased from GE Healthcare Life Sciences (Buckinghamshire, UK). Instant thin-layer chromatography-silica gel (ITLC-SG) plates were purchased from Agilent Technologies, Inc. (Santa Clara, CA, USA).

### Liposome preparation

A standard thin-film hydration method was used for liposome synthesis. DSPC, cholesterol, DSPE-PEG(2 k), and DSPE-PEG(2 k)-DBCO (in a molar ratio of 10.6:7.2:1:1) were dissolved in a mixture of chloroform and methanol (2:1, v/v). The mixture was evaporated under N_2_ gas until a thin lipid film was formed. Following evaporation, the lipid film was vacuumed for 4 h to remove any residual organic solvents from the lipid layer. The lipid film was hydrated with distilled water (1 mL) containing clodronate (20 mg, 55.4 µmol) and dispersed by vortexing and sonication. The liposomal solution was extruded through a polycarbonate track-etch (PCTE) membrane filter to obtain the desired size. The liposomes were ultrafiltered through Amicon Ultra 100 kDa filter centrifuge tubes at 5000 xg for 5 min. To obtain fluorescence (FI) conjugated liposomes, FNR648-N_3_ (7.67 µg, 10 nmol) or DiI (9.3 µg, 10 nmol) was added to the liposomes and incubated at 4 ℃. This conjugate was purified from free FI using a PD-10 column. For liposome conjugates with a targeting moiety, the targeting compound Man-N_3_ was added to half the amount of PEG. The conjugate was then purified using a PD-10 column.

### Characterization of liposomes

The hydrodynamic diameter and size distribution of liposomes diluted 50-fold in distilled water were measured using dynamic light scattering and nanoparticle tracking analysis (DLS and NTA, Malvern Instruments Ltd., Worcestershire, UK). The liposome morphology was observed using TEM (JEM-1400, JEOL, USA). To determine stability under physiological conditions, liposome stability tests were conducted in PBS, human serum, and cell media (DMEM) at different time points (0, 1, 7, and 14 days). The absorbance at 309 nm was measured using NanoDrop^®^ ND-1000 (NanoDrop Technologies, Wilmington, DE, USA) to confirm the absorbance peak of DBCO.

### Radiolabeling of liposomes and stability test

A vial containing ^64^Cu was dried using N_2_ gas in a fume hood for 30 min. After that, 200 µL of 1 M sodium acetate buffer (pH 5) was added to the vial to adjust the pH to 5. NOTA-N_3_ (10 µg, 18 nmol) dissolved in distilled water (10 µL) was added, and the mixture was heated at 70 ℃ for 5 min. Finally, 10 µL of [^64^Cu]Cu-NOTA-N_3_ was added to the liposomes in PBS and incubated overnight at 4 ℃. To remove the unchelated free ^64^Cu ions, the ^64^Cu-labeled liposomes synthesized through click chemistry were purified using a PD-10 column and eluted with PBS. Thin-layer chromatography was performed on ITLC-SG paper using citric acid (0.1 M) as the mobile phase to determine the radiolabeling efficiency. The *R*_f_ values of the free ^64^Cu, [^64^Cu]Cu-NOTA-N_3_, and ^64^Cu-labeled liposomes were 0.9 – 1.0, 0.7 – 0.8, and 0.0 – 0.1, respectively (Additional file 1: Fig. S8).

To demonstrate radiostability, the ^64^Cu-labeled liposomes were diluted tenfold with human serum. The radiolabeling efficiency of ^64^Cu-labeled liposomes dissolved in PBS and human serum was measured at different time point (0, 12, and 24 h), to confirm the stable conjugation of the radiolabeled agent during the imaging procedure.

### Determination of the clodronate encapsulation efficiency

In the ultrafiltration of liposomes using Amicon Ultra 100 kDa filter centrifuge tubes, the filtrate was collected [51]. The absorbance was measured at 205 nm to determine the concentration of clodronate in the solution. Finally, the EE% was calculated using the following equation:

$$\text{EE}\% = ~\frac{{{\text{Total}}\,{\text{amount}}\,{\text{of}}\,{\text{drug}} - {\text{unencapsulated}}\,{\text{drug}}~}}{{{\text{Total}}\,{\text{amount}}\,{\text{of}}\,{\text{drug}}}} \times 100$$

### Cell viability test

RAW264.7 cells (murine macrophages) were obtained from the Korean Cell Line Bank (Seoul, Korea). RAW264.7 cells were plated 100 mm diameter cell culture dish, 1×10^7^ cells/dish, and maintained routinely in DMEM with 10% FBS and 1% penicillin/streptomycin (PS). Cells were passaged at 70–80% confluence and harvested at passage numbers 7–12. Cells were seeded at 2 × 10^4^ cells onto 96-well plates and incubated at 37 ℃ for 24 h in a humidified incubator containing 5% CO_2_. After removal of media, liposomes with different concentration of clodronate (100, 200, and 400 µg/mL) were added to each well and the incubation was continued at 37 ℃ for 24 h. The control group comprised cells that were not treated with liposomes. After 24 h, the media was removed and CCK-8 reagent (Dojindo Molecular Technologies, Kumamoto, Japan) was added to each well followed by incubation at 37 ℃ for 2 h. The absorbance was measured at 450 nm using a GLOMAX Multi Detection System (Promega BioSystems, Sunnyvale, California, USA). The viability of cells was expressed as a percentage of the viability of the control cells.

### Cellular uptake study

RAW264.7 cells were maintained as described above and used for experiments at passage numbers 7–12. Cells were seeded at 1 × 10^5^ cells onto 12-well plate and incubated at 37 ℃ for 24 h. After the removal of media, liposomes with 200 µg/mL of clodronate were added to each well and incubated at 37 ℃ for 4 h. After incubation, the cells were washed thrice with DPBS; 4% PFA was added as a fixative, and the cells were incubated at 37 ℃ for 10 min. The cells were then washed thrice with DPBS, stained using mounting medium with DAPI (Vectashield, Vector Laboratories), and fixed on a glass slide using a cover glass. All observations were performed using a laser scanning confocal microscope (LSM800, Carl Zeiss, Oberkochen, Germany), with 648 nm laser excitation; the fluorescence was observed at a wavelength of 671 nm.

### In vivo PET imaging

Six-week-old male mice (C57BL/6) were purchased from Koatech (Pyeongtaek, South Korea). Approximately 1.85 MBq of ^64^Cu-labeled liposomes with clodronate (500 µg, 1.39 µmol) were injected through tail vein into seven-week-old normal mice (C57BL/6) anesthetized with 2% isoflurane, to confirm the in vivo biodistribution. The number of L and ML to be injected was determined based on the number of CL and CML injected. The PET scan images were acquired at different time points (0, 2, 8, and 24 h) after injection, using a preclinical PET/X-ray scanner (GENISYS4, Sofie Bioscience, California, USA). PET imaging was conducted using the InVivoScope software (version 2.0). The region of interest was calculated using the AMIDE software to quantitatively evaluate the uptake in the blood pool and liver. The time-activity curve was fitted based on % ID/g at each time point.

### Biodistribution analysis

The biodistribution of ^64^Cu-labeled liposomes was evaluated in normal mice (C57BL/6). Approximately 0.2 MBq of ^64^Cu-labeled liposomes with clodronate (500 µg, 1.39 µmol) was injected through the tail vein of seven-week-old normal mice. Clodronate-free liposomes (L and ML) were injected as described earlier. The animals were sacrificed by CO_2_ inhalation at different time points (0, 2, 8, and 24 h) following tail vein injection, and the various organs (blood, intestine, spleen, stomach, liver, kidney, heart, and lung) were dissected. Radioactivity was measured using an automatic gamma counter (Wizard, PerkinElmer, USA). Counts per minute were decay-corrected, and the results are expressed as % ID/*g*.

### Ex vivo tissue fluorescence imaging

DiI-labeled Clodrosome^®^ and m-Clodrosome^®^ (0.1 mL; 5 mg/mL) and DiI-labeled CL and CML with clodronate (500 µg, 1.39 µmol, 0.1 mL) were injected into normal mice (C57BL/6) through the tail vein. In addition, DiI-labeled L and ML were injected at the same particle number as DiI-labeled CL and CML. The animals were sacrificed by CO_2_ inhalation 24 h after injection, and their livers were dissected. The liver was embedded in the optimal cutting temperature compound (OCT compound) at -20 ℃. The livers were cut into 7 µm thick sections using a Leica CM1860 cryostat (Leica Biosystems, Wetzlar, Germany), and the sections were placed on glass slides. The liver sections were stained with mounting medium with DAPI, and they were covered with a cover glass. Fluorescence images were acquired using a LSM800 laser scanning confocal microscope.

### Preparation of tumor model

4T1 breast cancer-bearing mice were prepared for the evaluation of efficacy. 4T1 cells were cultured in vitro in RPMI-1640 with 10% FBS and 1% AA at 37 ℃ in a humidified incubator containing 5% CO_2_. 4T1 cells (5 × 10^5^ cells 100 µL^−1^ of normal saline) were injected into the right flank of normal mice (Balb/c-nude). Efficacy evaluation in 4T1-bearing mice was conducted when the implanted 4T1 tumor reached approximately 200 mm^3^. Tumor volume was calculated from the caliper measurements using the formula (width^2^ × length)/2 every 3 day.

### Immunohistochemistry

Normal mice (C57BL/6) were intravenously injected with 0.1 mL of 5 mg/mL Clodrosome^®^ and m-Clodrosome^®^, and 0.1 mL of CL and CML with clodronate (500 µg and 1.39 µmol, respectively). The number of injected liposomal particles for clodronate-free liposomes (L and ML) was the same as that for CL and CML. The animals were sacrificed by CO_2_ inhalation 24 h after injection and the livers were dissected. 4T1-bearing mice were intravenously injected with a single dose of the same amount of normal saline, CL, CML, Clodrosome^®^, or m-Clodrosome^®^ as that injected into normal mice. The animals were sacrificed by CO_2_ inhalation after 2 weeks of follow-up and dissected to identify tumors. Formalin-fixed and paraffin-embedded liver and tumor tissues were cut into 4 µm thick sections and automatically stained with a rabbit anti-CD206 antibody (1:1000, ab64693, Abcam), using the standard protocols on the Ventana Discovery XT automated immunohistochemistry system (Roche, Switzerland). The stained slides were imaged using a Leica SCN400F slide scanner (Leica Microsystems, Germany) at 400 × magnification. After immunohistochemistry, H&E staining was performed to observe the histological abnormalities.

### Statistical analysis

All statistical analyses were performed using the GraphPad Prism software (version 5.0) and presented as the mean ± standard deviation (SD). Means were compared using one-way analysis of variance, followed by Tukey’s post-hoc test. P-values < 0.05 were considered statistically significant and were represented by *P < 0.05; *P < 0.01; **P < 0.001; ***.

### Supplementary Information

**Additional file 1: Figure S1. **NTA analysis of Liposomes. The size distribution of (**a**) Clodrosome and m-Clodrosome and (**b**) liposome nanoplatforms in PBS was measured using the NTA system. **Figure S2.** Stability of liposomes at different physiological conditions (PBS, human serum, and cell media (DMEM). No visible aggregates or precipitates of liposomes were observed in any of the experimental groups after 14 days. **Figure S3. **Clodronate releasing test. The clodronate encapsulation efficiency of the liposomes was measured using a nanodrop. None of the groups showed significant differences. Statistical analysis was conducted using one-way analysis of variance. **Figure S4. **Cell viability test of RAW264.7 treated liposomes. Comparison of liposomes with Clodrosome^®^ and m-Clodrosome^®^. None of the groups showed significant differences. Statistical analysis was conducted using one-way analysis of variance. **Figure S5. **RAW264.7 cell uptake of liposomes. Comparison of the cellular uptake of liposomes at different time points (0.5, 1, 2, 4, and 24 h). All scale bars are 75 µm. **Figure S6.** Confocal images of the liver tissue treated with liposomes. Ex vivo tissue fluorescence images were acquired 24 h post-injection of liposomes in normal mice. All scale bars represent 250 µm. **Figure S7. **Histological analysis of H&E stained liposome-treated liver tissue. **Figure S8.** Labeling efficiency of all the liposomes. The labeling efficiency of all the liposomes used in the experiments was assessed using click chemistry with [^64^Cu]Cu-NOTA-N_3_. The radiochemical purity of all the liposomes was determined using the radio TLC chromatogram and percentage of value at *R*_f_ = 0.0–0.1.

## Data Availability

The datasets generated during and/or analyzed during the current study are available from the corresponding author on reasonable request.

## Abbreviations

L: Liposome
ML: Mannosylated liposome
CL: Clodronate-encapsulated liposome
CML: Clodronate-encapsulated mannosylated liposome
TAM: Tumor-associated macrophages
API: Active pharmaceutical ingredient
DDS: Drug delivery system
PDI: Polydispersity index
TME: Tumor microenvironment
SPAAC: Strain-promoted alkyne-azide cycloaddition
FI: Fluorescence
DLS: Dynamic light scattering
NTA: Nanoparticle tracking analysis
TEM: Transmission electron microscope
PBS: Phosphate buffered saline
DMEM: Dulbecco’s modified eagle medium
DPBS: Dulbecco’s phosphate buffered Saline
FBS: Fetal bovine serum
CCK-8: Cell counting kit-8
PFA: Paraformaldehyde
DAPI: 4ʹ6-Diamidino-2-phenylindole
FNR648-N_3_: Azido-Flamma 648
PET: Positron emission tomography
MPS: Mononuclear phagocytic system
DBCO: Dibenzocyclooctyne
CRD4: Carbohydrate recognition domain 4
ICI: Immune checkpoint inhibitors
