# Supplementary material for: Development of finely tuned liposome nanoplatform for macrophage depletion
Source: J Nanobiotechnology. 2024 Feb 29;22:83. doi: 10.1186/s12951-024-02325-7 (PMC10903058; doi:10.1186/s12951-024-02325-7)
Supplement: Supplementary file 1 — Additional file 1: Figure S1. NTA analysis of Liposomes. The size distribution of (a) Clodrosome and m-Clodrosome and (b) liposome nanoplatforms in PBS was measured using the NTA system. Figure S2. Stability of liposomes at different physiological conditions (PBS, human serum, and cell media (DMEM). No visible aggregates or precipitates of liposomes were observed in any of the experimental groups after 14 days. Figure S3. Clodronate releasing test. The clodronate encapsulation efficiency of the liposomes was measured using a nanodrop. None of the groups showed significant differences. Statistical analysis was conducted using one-way analysis of variance. Figure S4. Cell viability test of RAW264.7 treated liposomes. Comparison of liposomes with Clodrosome® and m-Clodrosome®. None of the groups showed significant differences. Statistical analysis was conducted using one-way analysis of variance. Figure S5. RAW264.7 cell uptake of liposomes. Comparison of the cellular uptake of liposomes at different time points (0.5, 1, 2, 4, and 24 h). All scale bars are 75 µm. Figure S6. Confocal images of the liver tissue treated with liposomes. Ex vivo tissue fluorescence images were acquired 24 h post-injection of liposomes in normal mice. All scale bars represent 250 µm. Figure S7. Histological analysis of H&E stained liposome-treated liver tissue. Figure S8. Labeling efficiency of all the liposomes. The labeling efficiency of all the liposomes used in the experiments was assessed using click chemistry with [64Cu]Cu-NOTA-N3. The radiochemical purity of all the liposomes was determined using the radio TLC chromatogram and percentage of value at Rf = 0.0–0.1. [file 12951_2024_2325_MOESM1_ESM.docx]

**Development of finely tuned liposome nanoplatform for macrophage depletion**

Tae Hyeon Choi^1,2†^, Ran Ji Yoo^1,3,4†^, Ji Yong Park^1,5^, Ji Yoon Kim^1,5^, Young Chan Ann^1,6^, Jeongbin Park^2^, Jin Sil Kim^1^, Kyuwan Kim^1^, Yu Jin Shin^1,7,8^, Yong Jin Lee^9^, Kyo Chul Lee^9^, Hyewon Chung^7,8,10^, Seung Hyeok Seok^7,8,10^, Hyung-Jun Im^2,5,7*^, Yun-Sang Lee^1,3,5,7,8*^

^1^Department of Nuclear Medicine, Seoul National University College of Medicine, Seoul, South Korea.

^2^Department of Molecular Medicine and Biopharmaceutical Sciences, Graduate School of Convergence Science and Technology, Seoul National University, Seoul, South Korea.

^3^Department of Nuclear Medicine, Seoul National University Hospital, Seoul, South Korea.

^4^Biomedical Research Institute, Seoul National University Hospital, Seoul, South Korea.

^5^Institute of Radiation Medicine, Medical Research Center, Seoul National University College of Medicine, Seoul, South Korea.

^6^School of Dentistry, Seoul National University, Seoul, South Korea.

^7^Cancer Research Institute, Seoul National University College of Medicine, Seoul, South Korea.

^8^Department of Biomedical Sciences, Seoul National University College of Medicine, Seoul, South Korea.

^9^Division of Applied RI, Korea Institute of Radiological and Medical Sciences (KIRAMS), Seoul, South Korea.

^10^Department of Microbiology and Immunology, and Institute of Endemic Disease, Seoul National University College of Medicine, Seoul, South Korea.

^†^These authors contributed equally.

**Corresponding authors**

Yun-Sang Lee, Ph.D.

Department of Nuclear Medicine, Seoul National University Hospital

101 Daehak-ro, Jongno-gu, Seoul, Republic of Korea.

Email: [wonza43@snu.ac.kr](mailto:wonza43@snu.ac.kr)

Hyung-Jun Im

Department of Molecular Medicine and Biopharmaceutical Sciences, Graduate School of Convergence Science and Technology, Seoul National University, Seoul 08826, Republic of Korea.

Email: iiihjjj@snu.ac.kr

**
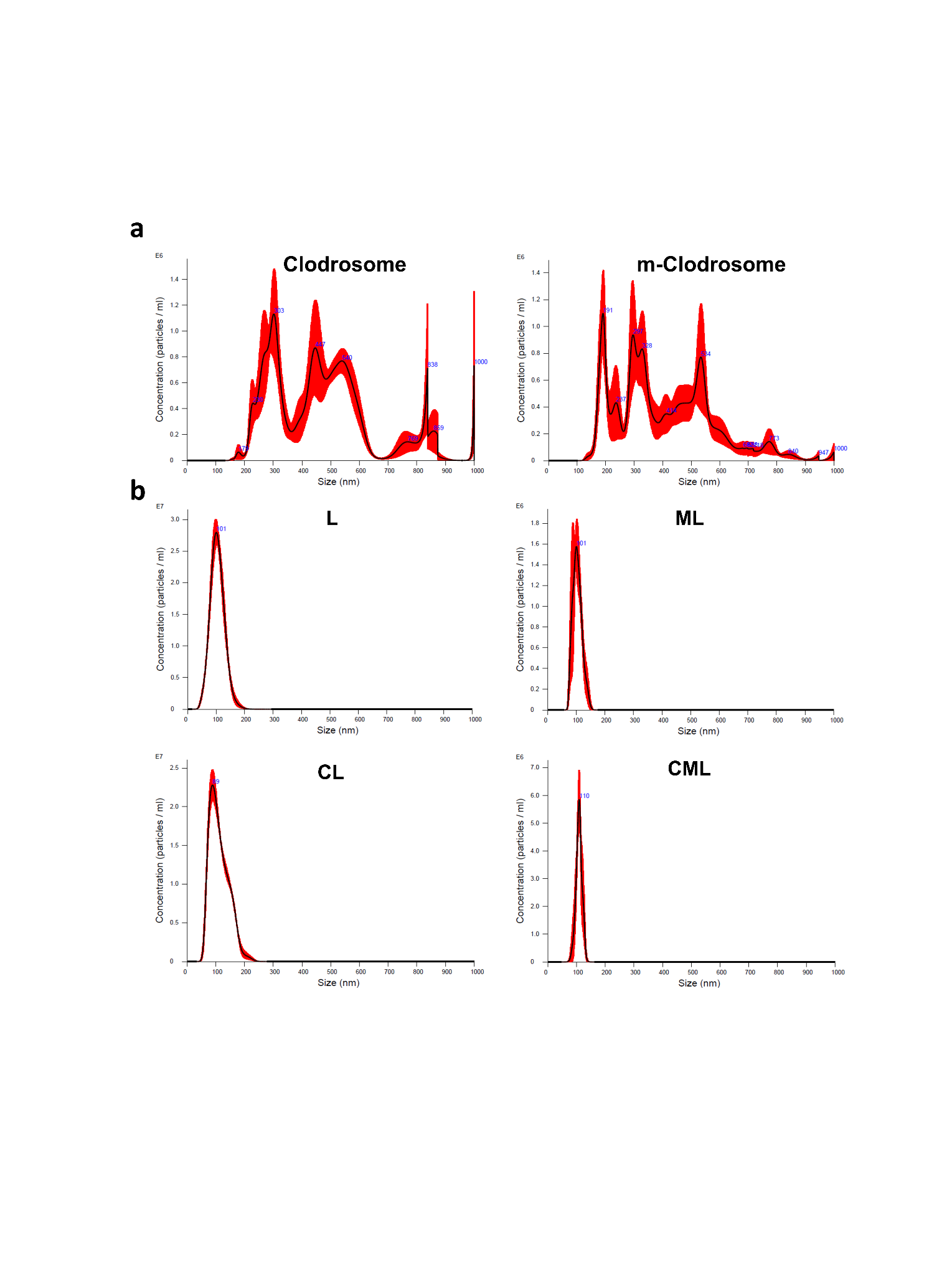
**

**Figure S1. NTA analysis of Liposomes.** The size distribution of (a) Clodrosome and m-Clodrosome and (b) liposome nanoplatforms in PBS was measured using the NTA system.

**
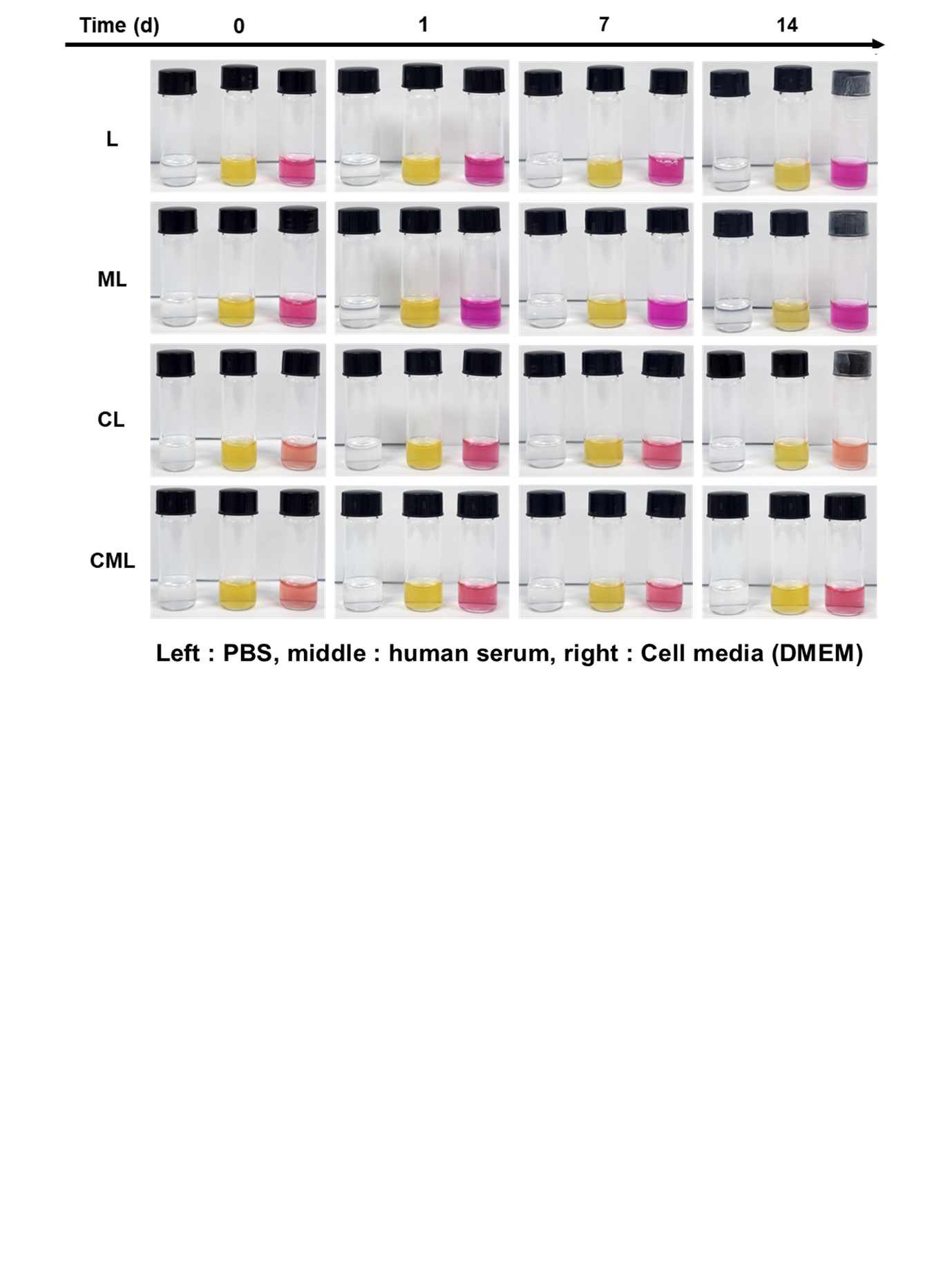
**

**Figure S2. Stability of liposomes at different physiological conditions (PBS, human serum, and cell media (DMEM).** No visible aggregates or precipitates of liposomes were observed in any of the experimental groups after 14 days.

**
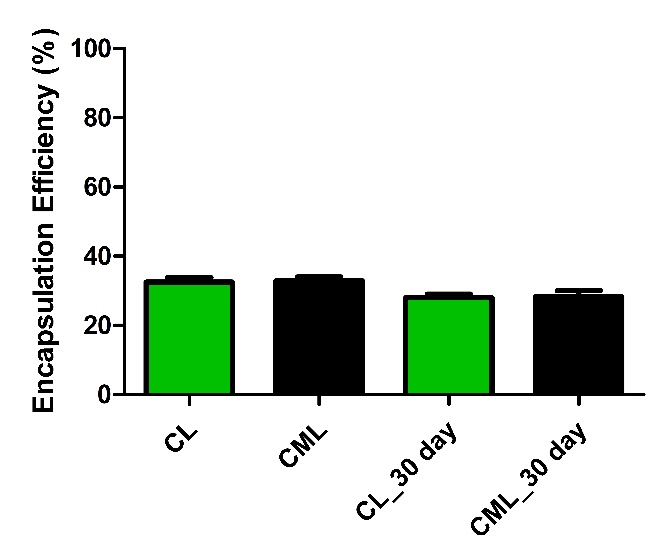
**

**Figure S3. Clodronate releasing test.** The clodronate encapsulation efficiency of the liposomes was measured using a nanodrop. None of the groups showed significant differences. Statistical analysis was conducted using one-way analysis of variance.

**
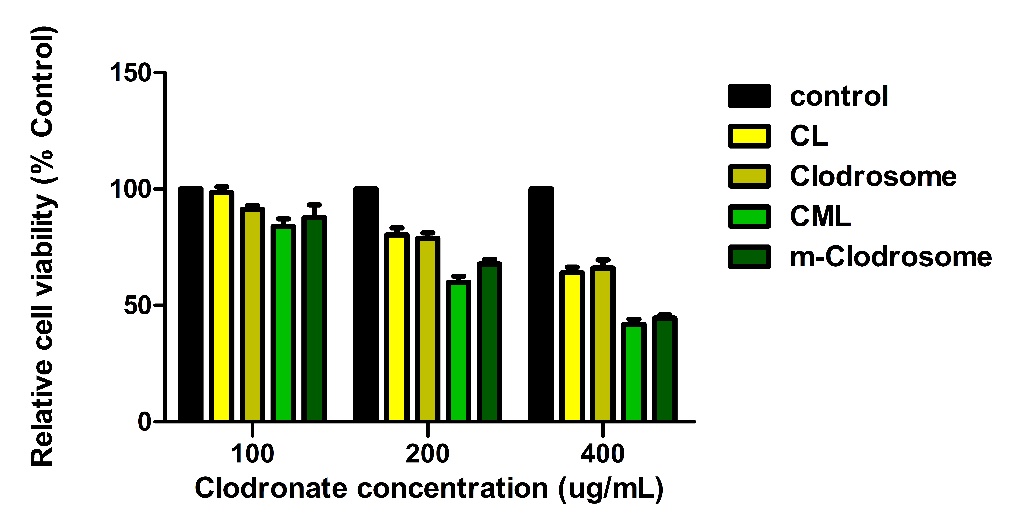
**

**Figure S4. Cell viability test of RAW264.7 treated liposomes.** Comparison of liposomes with Clodrosome^®^ and m-Clodrosome^®^. None of the groups showed significant differences. Statistical analysis was conducted using one-way analysis of variance.

**
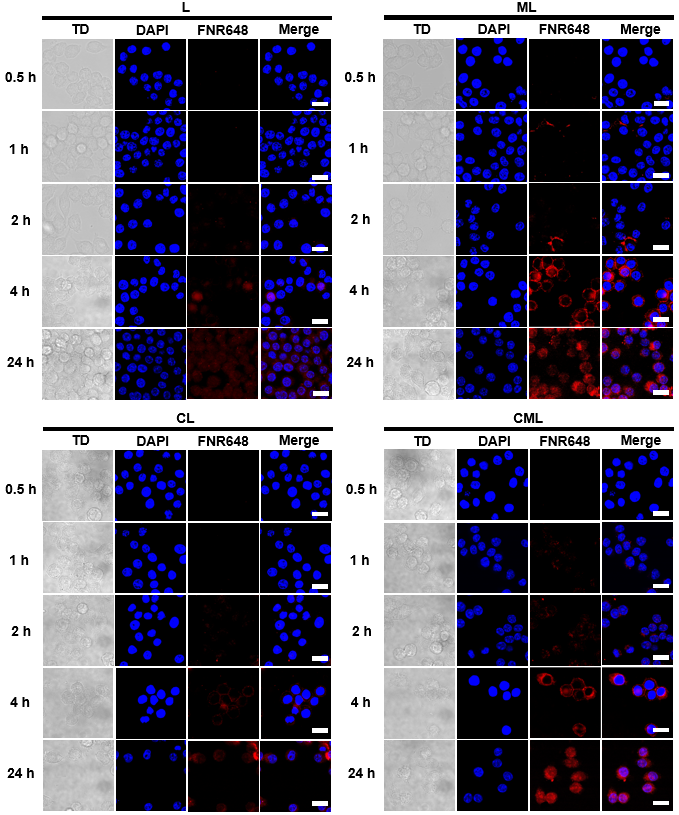
**

**Figure S5. RAW264.7 cell uptake of liposomes.** Comparison of the cellular uptake of liposomes at different time points (0.5, 1, 2, 4, and 24 h). All scale bars are 75 µm.

**
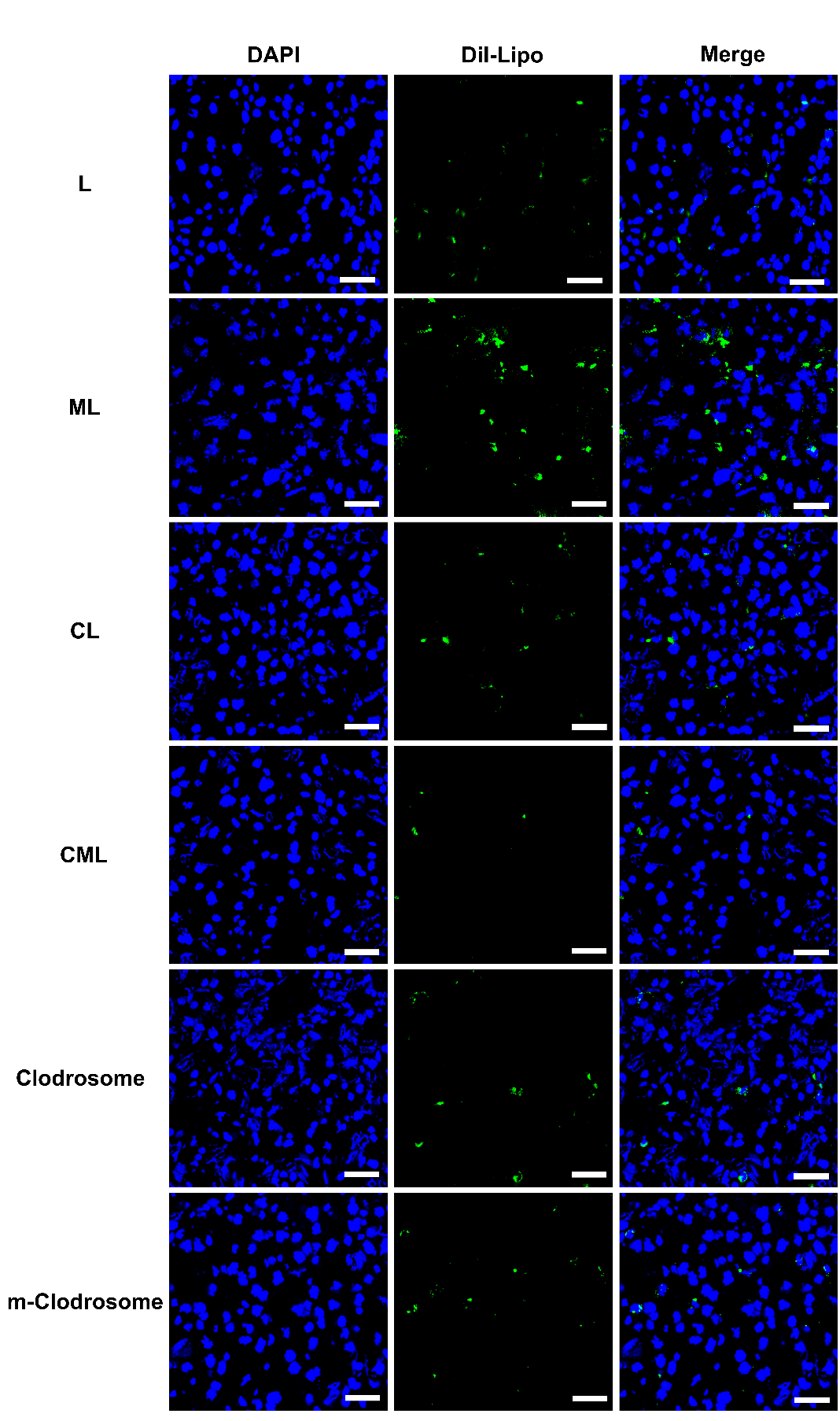
**

**Figure S6. Confocal images of the liver tissue treated with liposomes.** Ex vivo tissue fluorescence images were acquired 24 h post-injection of liposomes in normal mice. All scale bars represent 250 µm.

**
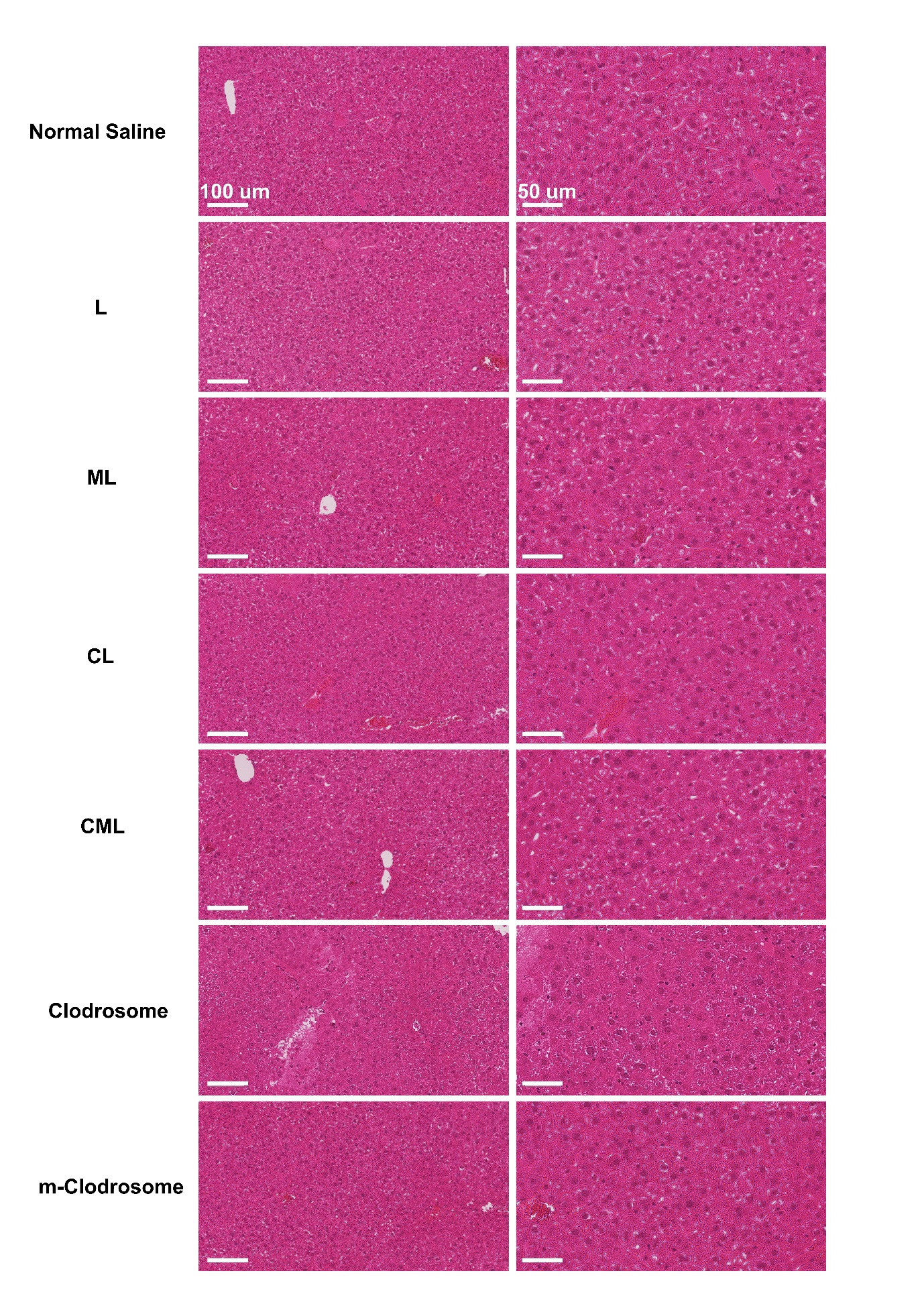
 Figure S7. Histological analysis of H&E stained liposome-treated liver tissue**

**
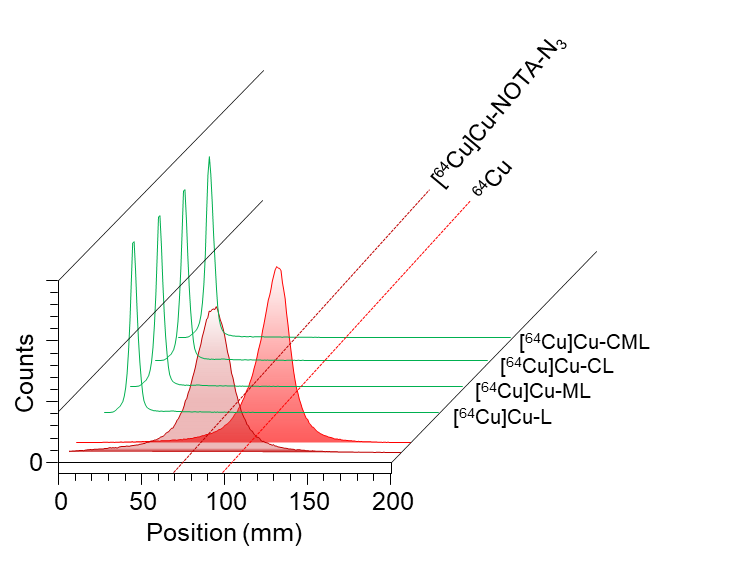
**

**Figure S8. Labeling efficiency of all the liposomes**. The labeling efficiency of all the liposomes used in the experiments was assessed using click chemistry with [^64^Cu]Cu-NOTA-N_3_. The radiochemical purity of all the liposomes was determined using the radio TLC chromatogram and percentage of value at *R*_f_ = 0.0~0.1.
